# Supplementary figures and images for: Temporal dynamics of bacterial and fungal communities during the infection of Brassica rapa roots by the protist Plasmodiophora brassicae
Source: PLoS One. 2019 Feb 25;14(2):e0204195. doi: 10.1371/journal.pone.0204195 (PMC6388920; doi:10.1371/journal.pone.0204195)

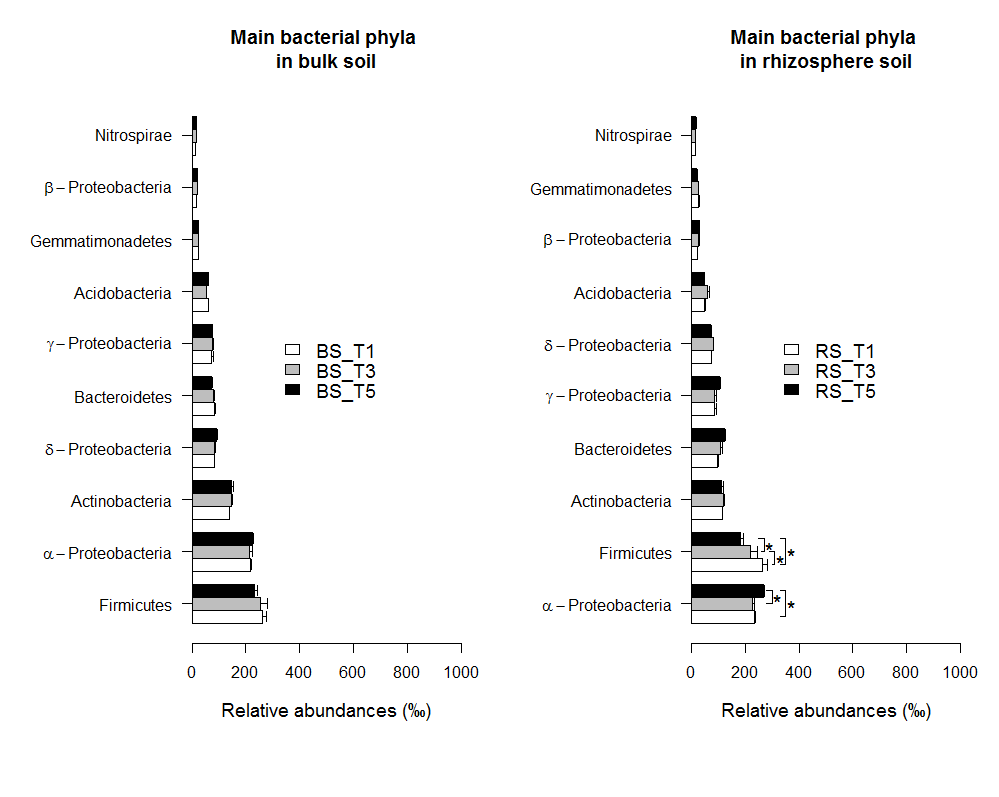

Supplement: S1 Fig — Richness (i.e. observed OTU) and diversity (i.e. Shannon index) of non-inoculated root (R), rhizosphere (RS) and bulk soil (BS) samples at different sampling dates are represented. Bacterial diversities were estimated with OTUs count data normalized by sample size and rarefied to 1,000 counts. Sampling date refers to 10 (T1), 24 (T3) and 45 (T5) days after sowing (DAS). For each sample, the number of replicates was n = 3. At each sampling date, lowercase letters indicate significant differences (p-values ≤ 0.05) between conditions, which were assessed by ANOVA followed by post hoc Tukey's HSD test. (TIF) [file pone.0204195.s001.tif]

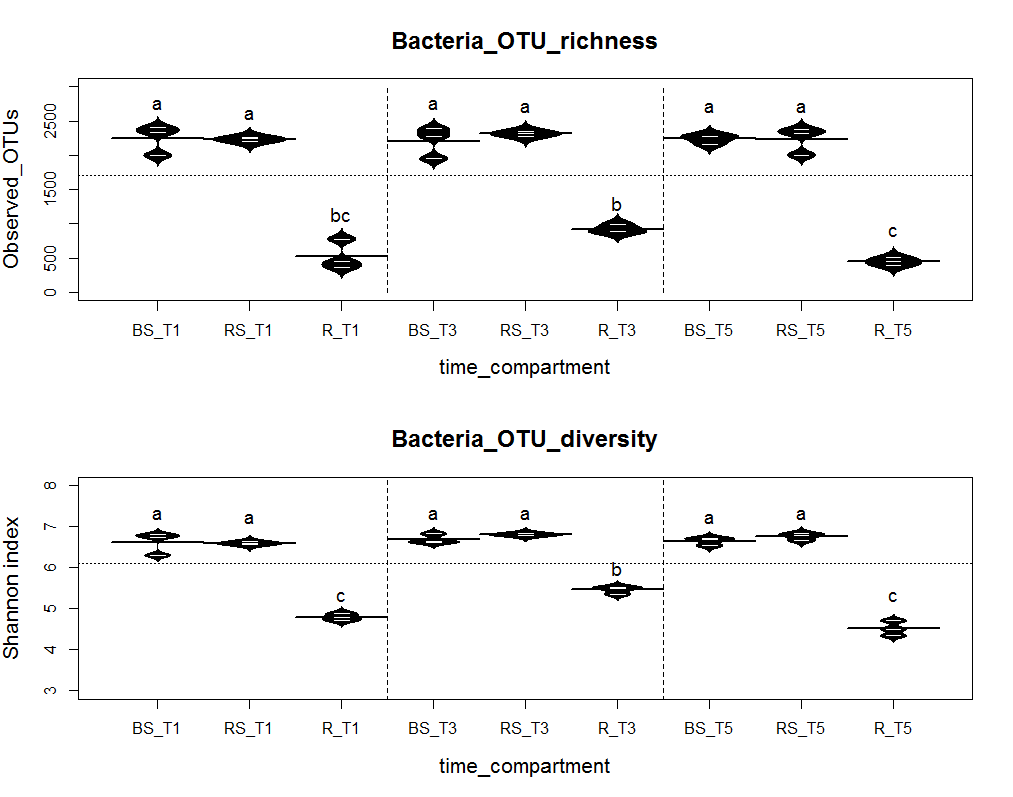

Supplement: S2 Fig — Richness (i.e. observed OTU) and diversity (i.e. Shannon index) of non-inoculated root (R), rhizosphere (RS) and bulk soil (BS) samples at different sampling dates are represented. Fungal diversities were estimated with OTUs count data normalized by sample size and rarefied to 1,000 counts. Sampling date refers to 10 (T1), 24 (T3) and 45 (T5) days after sowing (DAS). For each sample, the number of replicates was n = 3. At each sampling date, lowercase letters indicate significant differences (p-values ≤ 0.05) between conditions, which were assessed by ANOVA followed by post hoc Tukey's HSD test. (TIF) [file pone.0204195.s002.tif]

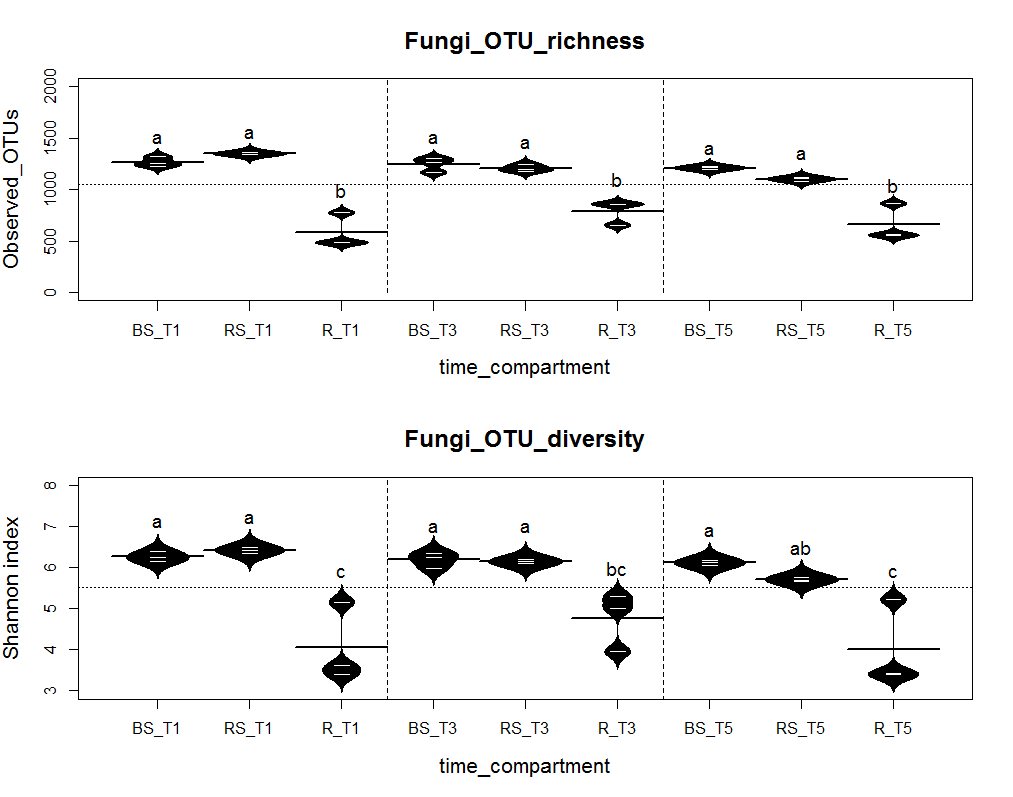

Supplement: S3 Fig — The variances explained by PCoA axes are given in parenthesis. Compartment refers to bulk soil (BS), rhizosphere soil (RS) and roots (R), represented by orange, brown and green colours, respectively. Sampling date refers to 10 (T1), 24 (T3) and 45 (T5) days after sowing (DAS). (TIF) [file pone.0204195.s003.tif]

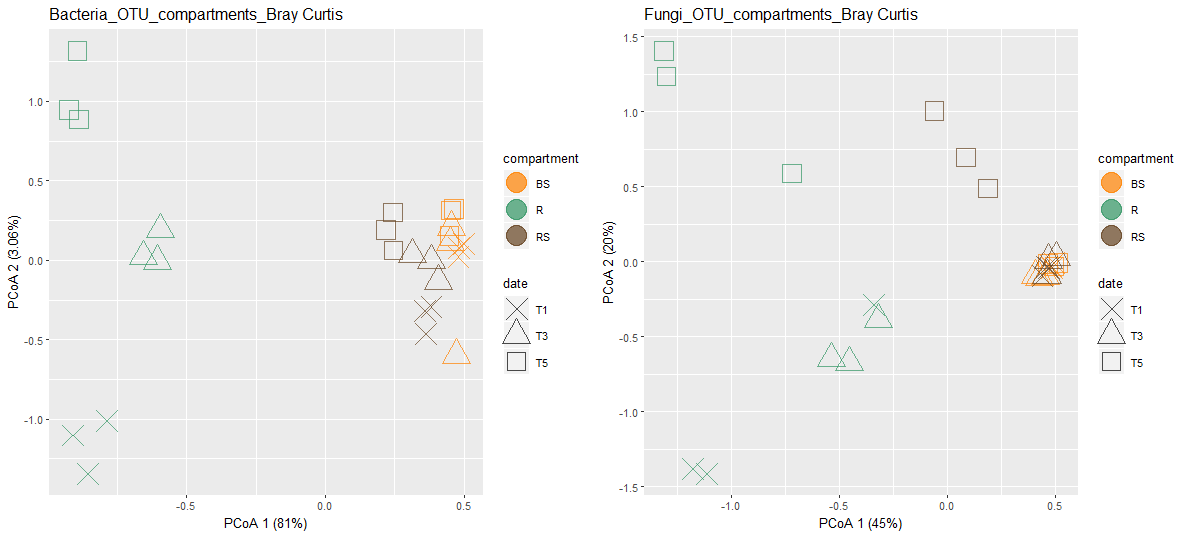

Supplement: S4 Fig — Compartment refers to bulk soil (BS), rhizosphere soil (RS) and roots (R), represented by orange, brown and green colours, respectively. Sampling date refers to 10 (T1), 24 (T3) and 45 (T5) days after sowing (DAS), represented by crosses, triangles and squares respectively. The variances explained by CPCoA axes are given in parenthesis. For each CPCoA, variations between samples in Bray-Curtis distances were constrained by compartment (in the left column) or sampling date (in the right column) factor. Canonical analysis of principal coordinates (CAP) was performed to quantify the influence of these factors on the β-diversity. The percentage of variation refers to the fraction of the total variance of the data explained by each constrained factor. The p-values indicate if the influence of each of these constrained factors on the β-diversity was significant (p-values ≤ 0.05). (TIF) [file pone.0204195.s004.tif]

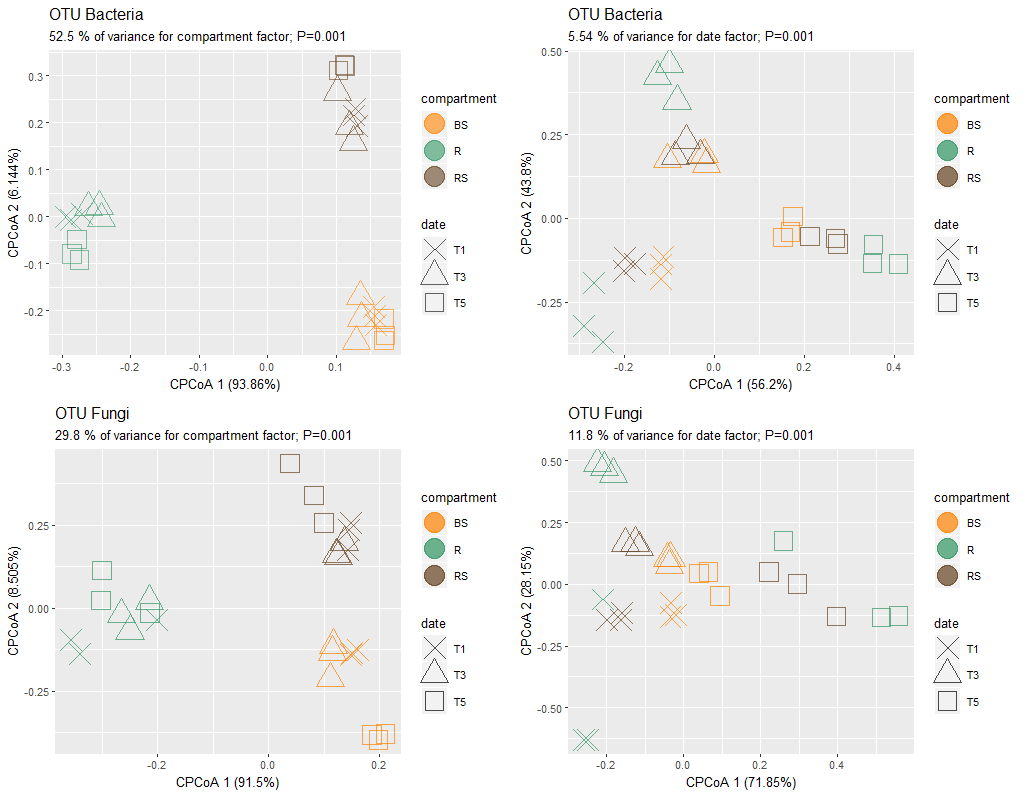

Supplement: S5 Fig — Mean values of relative abundances (expressed in ‰) were obtained from three replicates per compartment and sampling date. Sampling dates refer to 10 (T1), 24 (T3) and 45 (T5) days after sowing. The 10 main phyla-subphyla were represented. The Proteobacteria phylum was divided into four subphyla: α-, β-, γ- and δ-Proteobacteria. (TIF) [file pone.0204195.s005.tif]

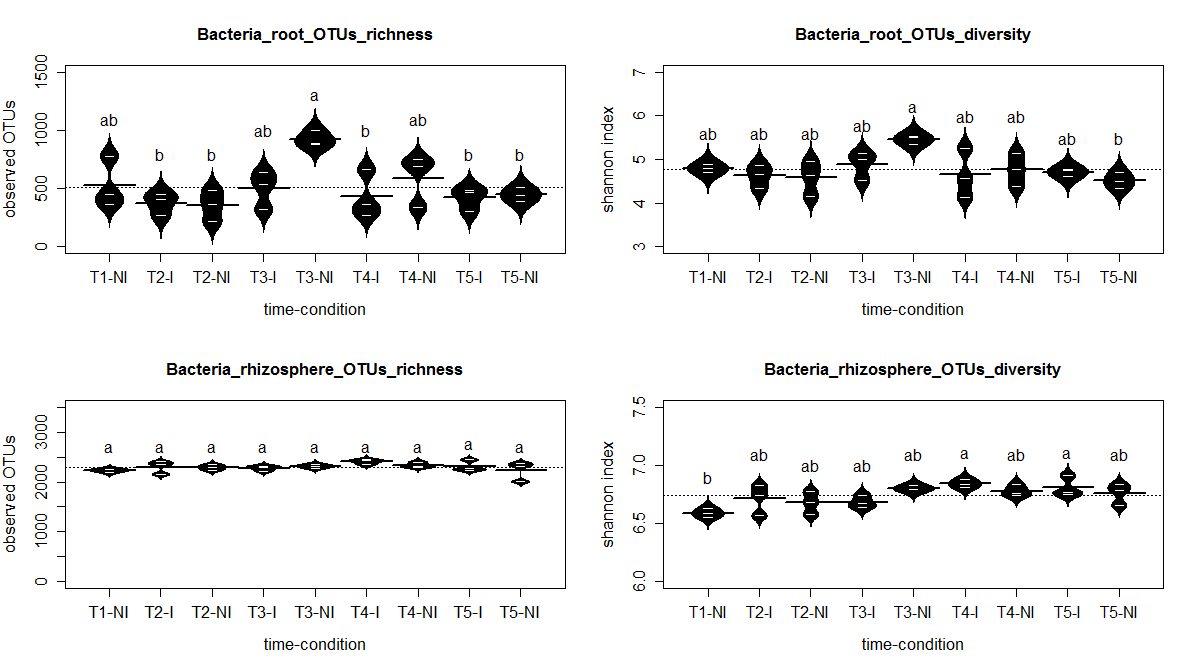

Supplement: S6 Fig — Richness (i.e. observed OTU) and diversity (i.e. Shannon index) of root (R) and rhizosphere (RS) samples from NI and I plants were measured at different sampling dates. Bacterial diversity was estimated with OTUs count data normalized by sample size and rarefied to 1,000 counts. Richness and diversity associated NI plants and plants inoculated by P. brassicae (I) at each sampling date (T1 to T5) were compared. Sampling date refers to 0 (T1), 7 (T2), 14 (T3), 23 (T4) and 35 (T5) days after inoculation (DAI) with P. brassicae. At each sampling date, lowercase letters indicate significant differences (p-values ≤ 0.05) between conditions, which were assessed by ANOVA followed by post hoc Tukey's HSD test. (TIF) [file pone.0204195.s006.tif]

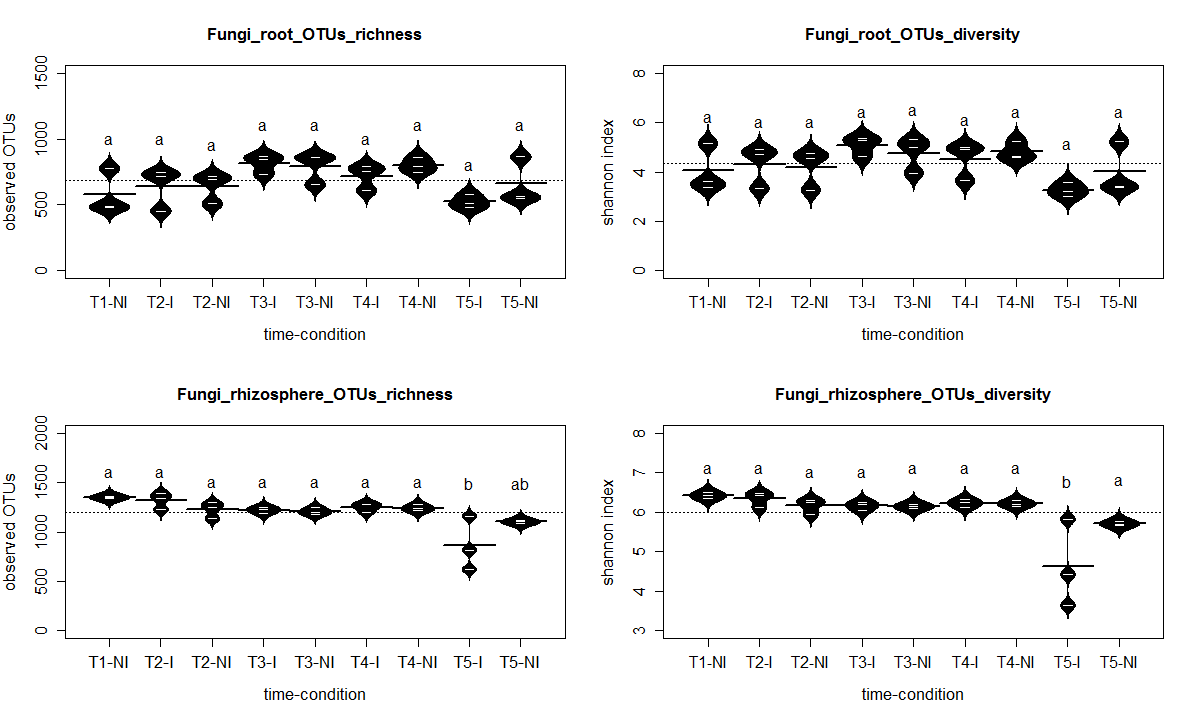

Supplement: S7 Fig — Richness (i.e. observed OTU) and diversity (i.e. Shannon index) of root (R) and rhizosphere (RS) samples from NI and I plants were measured at different sampling dates. Fungal diversity was estimated with OTUs count data normalized by sample size and rarefied to 1,000 counts. Richness and diversity associated to NI and I plants at each sampling date (T1 to T5) were compared. Sampling date refers to 0 (T1), 7 (T2), 14 (T3), 23 (T4) and 35 (T5) days after inoculation (DAI) with P. brassicae. At each sampling date, lowercase letters indicate significant differences (p-values ≤ 0.05) between conditions, which were assessed by ANOVA followed by post hoc Tukey's HSD test. (TIF) [file pone.0204195.s007.tif]
