# Supplementary material for: Temporal dynamics of bacterial and fungal communities during the infection of Brassica rapa roots by the protist Plasmodiophora brassicae
Source: PLoS One. 2019 Feb 25;14(2):e0204195. doi: 10.1371/journal.pone.0204195 (PMC6388920; doi:10.1371/journal.pone.0204195)
Supplement: S2 Table — The number of leaves per plant, the shoot and root fresh weight, the plant leaf areas, the plant height and root length were measured during the kinetics of plant growth at 10 (T1), 17 (T2), 24 (T3), 33 (T4) and 45 (T5) days after sowing, corresponding to 0 (T1), 7 (T2), 14 (T3), 23 (T4) and 35 (T5) days after inoculation. At each sampling date, numbers in bold and lowercase letters indicate significant differences (p-values ≤ 0.05) between inoculated (I) and non-inoculated (NI) plants. SEM: standard error of the mean; nd: not determined. (DOCX) [file pone.0204195.s009.docx]

**S2 Table**

|  |  |  |  |  |  |  |  |  |  |
| --- | --- | --- | --- | --- | --- | --- | --- | --- | --- |
|  | T1 | T2 | | T3 | | T4 | | T5 | |
|  | NI / I | NI | I | NI | I | NI | I | NI | I |
| Number of leaves per plant (± SEM) | 1.9 ± 0.3 | 3.5 ± 0.7 | 3.6 ± 0.9 | 6.4 ± 0.8 | 6.7 ± 0.6 | 8.3 ± 1.1 | 8.9 ± 1.4 | 11.5 ± 0.9 | 11.3 ± 1.7 |
| Plant height (cm ± SEM) | 8.4 ± 1.9 | 17.1 ± 2.5 | 17.8 ± 2.3 | 24.1 ± 2.3 | 24.9 ± 1.4 | 25.8 ± 2.1 | 27.4 ± 2.6 | 28.6 ± 3.0 | 27.6 ± 2.8 |
| Fresh shoot biomass (g ± SEM / plant) | 0.6 ± 0.2 | 3.1 ± 1.5 | 3.3 ± 1.5 | 10.5 ± 2.7 | 12.4 ± 1.5 | 22.1 ± 4 | 23.6 ± 8.7 | 34.2 ± 4.3 | 32.2 ± 9.2 |
| Plant leaf area (cm^2^ ± SEM / plant) | 7.9 ± 4.0 | 65.5 ± 31.4 | 72.1 ± 34.2 | 232.6 ± 69.5 | 258 ± 24.2 | 444.2 ± 104.5 | 503.3 ± 145.2 | 664.4 ± 103.4 | 635.5 ± 167.5 |
| Hypocotyl diameter (mm ± SEM) | 1.0 ± 0.1 | 1.6 ± 0.4 | 2.0 ± 0.5 | **2.3 ± 0.7** **^a^** | **3.0 ± 0.8 ^b^** | **3.7 ± 0.6** **^a^** | **7.6 ± 2.8 ^b^** | **4.9 ± 0.9** **^a^** | **10.6 ± 0.9** **^b^** |
| Root length (cm ± SEM) | nd | 19.7 ± 3.2 | 21.5 ± 5.0 | 26.9 ± 3.5 | 27.9 ± 2.8 | 29.1 ± 3.8 | 27.8 ± 3.9 | **31.7 ± 3.9 ^a^** | **23.4 ± 4.2** **^b^** |
| Fresh root biomass (g ± SEM / plant) | 0.14 ± 0.07 | 0.4 ± 0.3 | 0.3 ± 0.2 | 1.3 ± 0.6 | 1.7 ± 0.5 | 3.4 ± 1.6 | 3.8 ± 1.4 | **4.1 ± 1.0** **^a^** | **7.8 ± 2.5** **^b^** |
